# Compact and automated eDNA sampler for in situ monitoring of marine environments

**DOI:** 10.1038/s41598-023-32310-3

**Published:** 2023-03-30

**Authors:** Andre Hendricks, Connor M. Mackie, Edward Luy, Colin Sonnichsen, James Smith, Iain Grundke, Mahtab Tavasoli, Arnold Furlong, Robert G. Beiko, Julie LaRoche, Vincent Sieben

**Affiliations:** 1 Department of Electrical and Computer Engineering, Dalhousie University, Halifax, NS Canada; 2 Dartmouth Ocean Technologies Inc, Dartmouth, NS Canada; 3 Department of Pharmacology, Dalhousie University, Halifax, NS Canada; 4 Faculty of Computer Science, Dalhousie University, Halifax, NS Canada; 5 Department of Biology, Dalhousie University, Halifax, NS Canada

**Keywords:** Environmental impact, Electrical and electronic engineering, Genetics, Microbiology, Ocean sciences

## Abstract

Using environmental DNA (eDNA) to monitor biodiversity in aquatic environments is becoming an efficient and cost-effective alternative to other methods such as visual and acoustic identification. Until recently, eDNA sampling was accomplished primarily through manual sampling methods; however, with technological advances, automated samplers are being developed to make sampling easier and more accessible. This paper describes a new eDNA sampler capable of self-cleaning and multi-sample capture and preservation, all within a single unit capable of being deployed by a single person. The first in-field test of this sampler took place in the Bedford Basin, Nova Scotia, Canada alongside parallel samples taken using the typical Niskin bottle collection and post-collection filtration method. Both methods were able to capture the same aquatic microbial community and counts of representative DNA sequences were well correlated between methods with R$^{2}$ values ranging from 0.71–0.93. The two collection methods returned the same top 10 families in near identical relative abundance, demonstrating that the sampler was able to capture the same community composition of common microbes as the Niskin. The presented eDNA sampler provides a robust alternative to manual sampling methods, is amenable to autonomous vehicle payload constraints, and will facilitate persistent monitoring of remote and inaccessible sites.

## Introduction

Increasing human activity in aquatic environments has led to concerns over anthropogenic effects causing issues such as hypoxia, ocean acidification, and eutrophication caused by increased nutrient loading^1^. These impacts can impede growth of certain organisms such as calcifying marine species, whose shells and skeletons can be affected by acidification^2^ and promote the growth of other species including those that cause harmful algal blooms (HABs) which harm fish as well as the human economy^3,4^. The timescale of these changes and their consequent impacts can range from hours to years, and since each ecosystem is unique, changes can be difficult to track, requiring time-resolved *in situ* observations in order to properly assess changes.

Biological monitoring programs have traditionally focused on manual identification of key taxonomic groups of interest; however, these programs can be time consuming and require special training in taxonomic identification. In recent years, with a decrease in the cost of DNA sequencing and the increasing size of nucleic acid databases, environmental DNA (eDNA) is increasingly being used as a proxy for biodiversity in biological monitoring programs^5^. Monitoring eDNA involves studying all DNA present in the environment^6^ and is advantageous in multiple ways as it is non-invasive, and widely applicable to microbiota and metazoans alike using a rapidly evolving suite of analytical methods from sensitive DNA extraction to detection of unique barcode sequences^7^. There are numerous studies that have demonstrated the value of eDNA to study microbial diversity, given the importance of their role in primary production by phytoplankton and biogeochemical cycling of dead organic matter. For example, biomonitoring of microbiota in aquaculture settings has demonstrated the usefulness of eDNA to detect the rapid microbial response to environmental disturbance and assess management strategies for a sustainable aquaculture industry^8–10^. Furthermore, an increasing number of studies have demonstrated the important role that eDNA is destined to play for environmental monitoring of fish biodiversity^11^, tracking of marine mammals^12^ and other aspects of conservation biology^13^.

Current methods for eDNA sampling are often labour intensive, involving the collection of samples using Niskin bottles or similar equipment, followed by separate filtration and preservation steps, often using a peristaltic pump and freezer respectively. The manual components of eDNA sampling and analysis limit its use in remote settings, or in settings where regular samples must be taken, and require a trained individual to perform the process. Extending the applicability of eDNA methods to more-challenging problems requires automation, including the development of automated sampling equipment. Recently developed samplers range from single-filter systems to more-complex multi-filter systems, with each varying in parameters such as deployment duration, maximum depth rating, and chemicals/preservatives used. A representative list of current eDNA samplers, both commercially available and research prototypes, is outlined in Table 1.

**Table 1:** eDNA samplers in the literature and online.

Year	Instrument	Organization	Depth	Number of filters	Filter	Preservation	Self cleaning
Handheld sampler (not submersible)							
2018	eDNA Sampler^14^	Smith-Root	Surface	1	47 mm Filter	Y$^{1}$	N$^{2}$
Single filter sampler							
2014	Continuous Low-Level Aquatic Monitoring (C.L.A.M)^15^	Aqualytical	6.1 m	1	47 mm SPE disk	N	N
2021	Subsurface Automated Sampler for eDNA (SASe)^16^	National Oceanic and Atmospheric Administration (NOAA)	55 m	1	Sterivex Filter 0.22 $\upmu$m	Y	N
Multi-filter sampler							
2008	Phytoplankton Sampler (PPS)^17^	McLane research laboratories	5500 m	24	47 mm Filter	Y	Y
2012	Modular Autonomous Biosampler (MAB)^18^	Cellula Robotics Ltd.	200 m	200	47 mm Filter	Y	Y
2015	Environmental Sample Processor (ESP) Gen 3^19,20^	Monterey Bay Aquarium Research Institute (MBARI)	300 m	60	25 mm Durapore Filter 0.22 $\upmu$m	Y	Y
2019	in situ Autonomous Biosampler (IS-ABS)^21^	CIIMAR	150 m	16	Sterivex Filter 0.2 $\upmu$m	Y	Y$^{3}$
2020	PolyWAG (Water Acquired Genomics)^22^	Oregon State University	-	24	47 mm Filter Disc	Y	Y
2022	Large Volume eDNA Sampler^23^	Woods Hole Oceanographic Institution (WHOI)	6000 m	12	PES Filter 0.2 $\upmu$m	N	N
2022	eDNA Sampler	Dartmouth Ocean Technoogies Inc.(DOT)	20 m 3000 m$^{4}$	9	25 mm Filter	Y	Y

$^{1}$ Requires self-preserving filter.

$^{2}$ Sterilized Filter is provided but lacks acid cleaning protocol.

$^{3}$ Cleaning is performed with in situ water.

$^{4}$ Deep-rated unit is pressure compensated, filled with mineral oil.

Starting from the most advanced, the Environmental Sample Processor (ESP) houses an impressive 60 filter cartridges; however, the ESP samplers have remained primarily in research studies without fully transitioning to commercial workflows and applications, such as use by aquaculture operators, deployment in city harbours, wind turbine installations, etc. This sampler is based on an evolution of the pioneering ESP, an in situ sampler and DNA analyzer developed by the Monterey Bay Aquarium Research Institute (MBARI). These fully submersible analyzers were developed from 2001 to 2009^24^ and were termed Generation 1 and Generation 2. These were comprehensive labs-under-the-sea that performed sample collection and DNA extraction to feed PCR microfluidic devices, hybridization arrays, and sandwich assays. However, the ESP Generation 1 and Generation 2 instruments cost several hundred thousand dollars and are highly complex to deploy and service, with final contracts typically in the millions of dollars. The latest generation of instrumentation from MBARI, Generation 3, completely removed the integrated analysis, aiming to perform sample collection with preservation on underwater vehicles, followed by land-based laboratory genomics analysis^20^.

Even though cost and complexity were reduced for the ESP Generation 3, new and streamlined samplers aimed to further improve scalability for collecting eDNA in situ, including: the Subsurface Automated Sampler for eDNA (SASe), PolyWAG (Water Acquired Genomics), and the CLAM (Continuous Low-Level Aquatic Monitoring). These systems are priced in the thousands of dollars, thus making automated eDNA sampling more accessible. Most of these streamlined samplers have a single filter, tend not to carry preservation or cleaning reagents, and are suitable for short-term (hourly, daily) deployments. While some have longer-term deployment capability (SASe unit has onboard preservation capability), many lack the ability to self-clean with acids or bleach, nor do they flush the sample inlet/intake to minimize biofouling and cross-contamination. The polyWAG system offers 24 filters with integrated self-cleaning using air and self-preservation using ethanol. The added automation increases cost to 3000–5000 USD. Furthermore, these designs seldom consider form factors that are amenable toward integration with platforms or autonomous vehicle payload constraints, in some cases with exposed tubing and unfastened wires and electronics.

**Figure 1:**
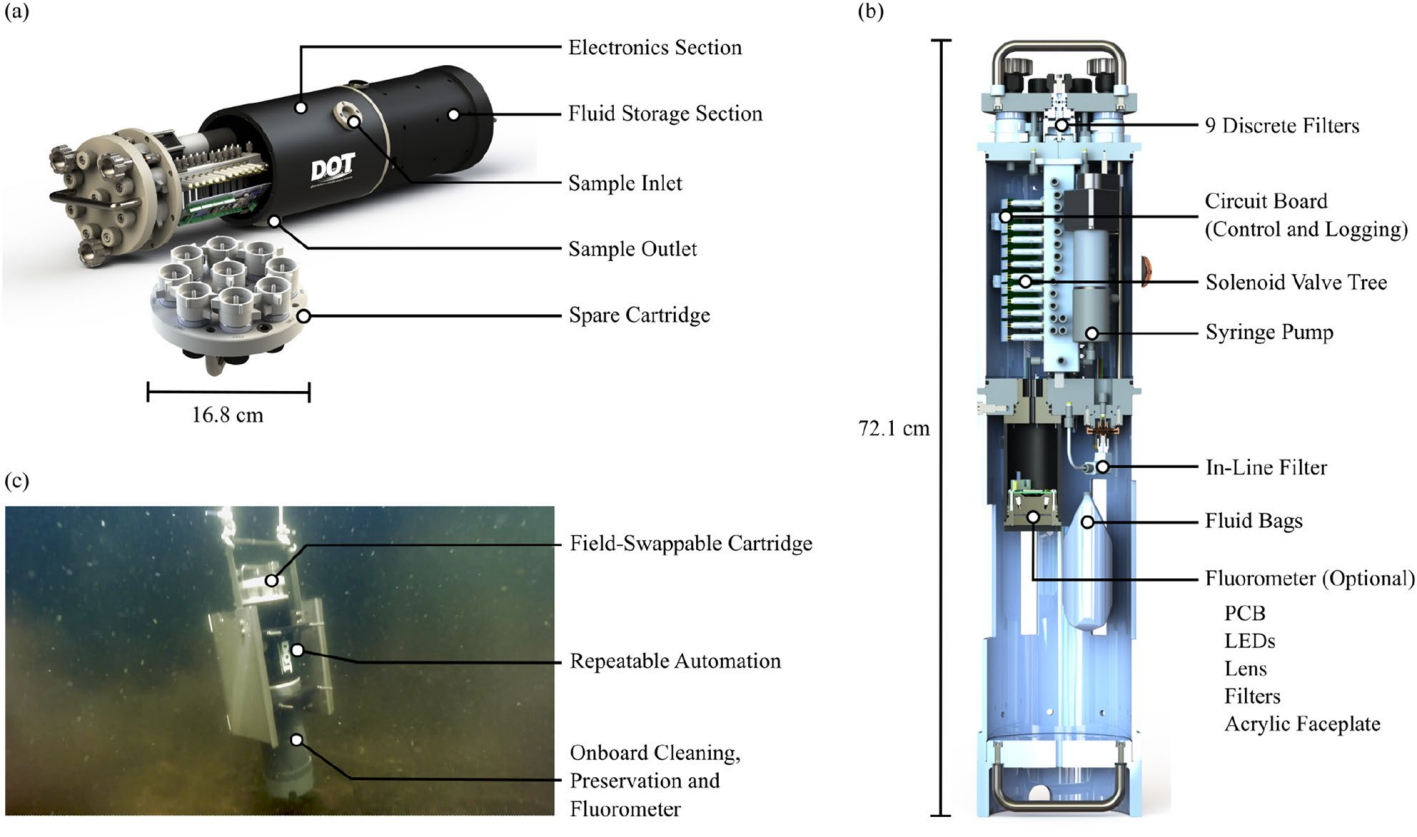
(**a**) 3D CAD rendering of the DOT eDNA sampler with partially exposed electronics section. (**b**) Cross-sectional view of the DOT eDNA sampler, highlighting all compartments, fluid storage bags and attached fluorometer. (**c**) Fully built DOT eDNA sampler deployed underwater.

Here we introduce a novel, autonomous eDNA sampler capable of collecting, filtering, and preserving a water sample using an innovative design, all within a single instrument, shown in Figure 1. The sampler can collect up to 9 discrete samples per deployment, which, unlike other samplers, are stored on a removable cassette that is easily changeable on site in less than 5 minutes. This allows for immediate redeployment of the instrument, where a new cassette can rapidly be loaded and the filled cassette is either analyzed in the field or back at the lab. Furthermore, the unit is self-cleaning to prevent biofouling, and all tubing is contained to the instrument housing to prevent snags on the lines during deployments. The sampler is also compact and designed with dual handles, making it able to be transported and deployed by a single person. The sampler has been made commercially available to purchase through Dartmouth Ocean Technologies Inc., Canada. The initial market price of the eDNA sampler is 55,000 USD for the instrument and 5000–10,000 USD in reagents, options, and additional filter cassettes. Here we describe the initial testing of the sampler in a real-world deployment off a small vessel. The user-focused design of the sampler allows for the standardization and simplification of eDNA collection, thereby improving sampling reliability and repeatability for environmental monitoring. Our sampler was able to match results obtained through the use of the typical Niskin bottle capture and peristaltic filtration methods from a microbial-community level down to the individual sequence level.

## Method and design

### System overview

Dalhousie University has collaborated with Dartmouth Ocean Technologies, Inc. (DOT) to create a novel eDNA sampler that features a simple modular approach that has three detachable sections: filter cartridge, electronics section, and fluid storage section, shown in Fig. 1. The fully assembled unit has a length of 72.1 cm and a width of 16.8 cm, weighing 11.3 kg in air and 3.3 kg in salt water. It is capable of cleaning between sample captures, preservation of captured samples and has 9 discrete filters, each 25 mm in diameter. Different filter membranes can be loaded into the filter holders (Advantec 43303010, Polypropylene), thus allowing for a wide variety of membrane materials and pore sizes to be used based on targeted species. The eDNA sampler’s filter cartridge is made from Polyetheretherketone (PEEK) material and holds the 9 filter holders. Once the filter cartridge is loaded with clean filters it can be attached to the electronics section of the eDNA sampler. This fast swap approach allows for multiple filter cartridges to be prepared and then loaded into the sampler as needed. The filter cartridge is secured by 3 knobs that are indexed to the electronic section to avoid assembly error. The version of the sampler used in this paper is depth rated to 20 m. However, a 3000 m version is available and has been successfully tested in a pressure chamber at ESL labs (Dartmouth, NS, Canada).

The eDNA sampler’s electronics section is the core of the instrument. It houses a pump and custom valve tree, along with a custom printed circuit board (PCB) for automation and data logging. The PCB and software will be described in the System Architecture section below. The valve tree consists of the fluid routing manifold, a pressure sensor, tubing inter-connections, and solenoid valves for the sampler. The valve tree also has ports that are used to fluidically couple to the filters on the filter cartridge, and to access the fluid bags loaded with reagents and stored in the fluid section of the sampler.

The eDNA sampler’s fluid storage section houses and protects all the required fluids and an optional fluorometer. The fluids stored in this section are as follows: 5% hydrochloric acid (HCl) (cleaning), RNAlater (preservation), purified Milli-Q water (rinsing) and waste. The fluids are stored in 100 and 500 mL Labtainer$^{\textrm{TM}}$ BioProcess Containers (BPC) and connected to the Electronics section with 1/4– 28 ports. The waste bag is used to hold chemicals that are deemed not safe to flush into the ocean or surrounding waters. RNAlater is used to preserve the collected samples, 5% HCl is used to clean the system fluid lines and backflow the sample inlet, and Milli-Q is used to flush the system between protocol steps. The 5% HCl and Milli-Q are effective at reducing cross-contamination that might take place in the system tubing and manifolds between sampling events. HCl and RNAlater used in this study were of analytical grade and supplied by Fisher Chemical (Waltham, MA, USA).

### Automation protocol

**Figure 2:**
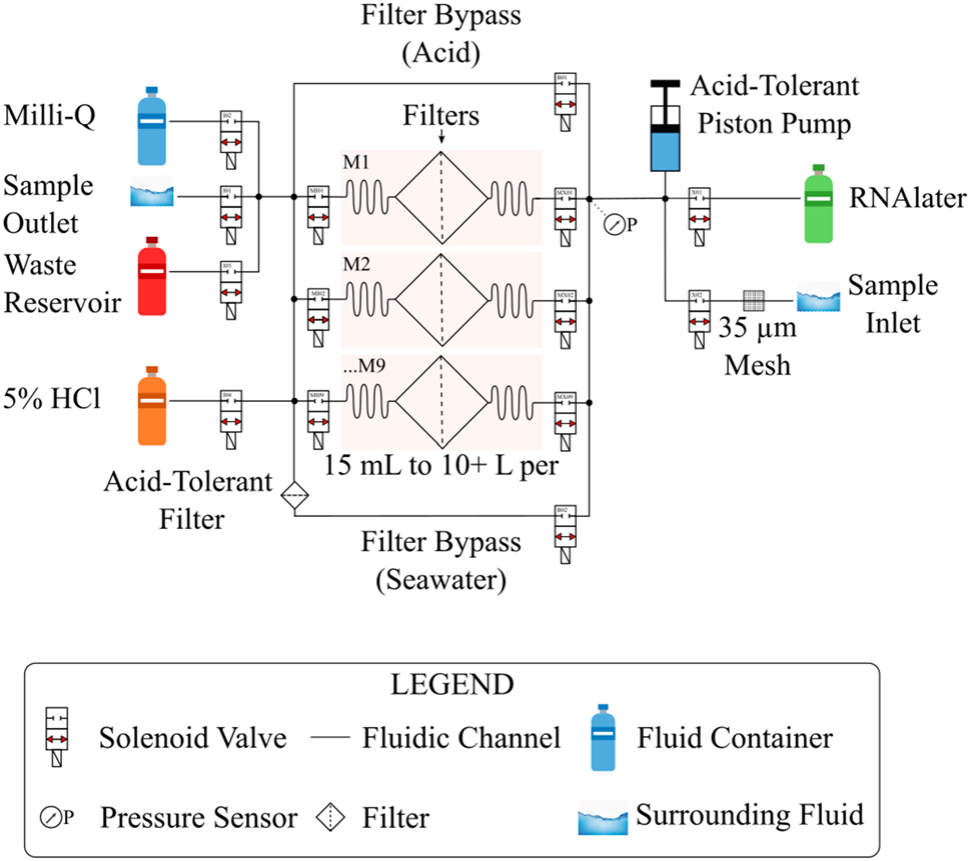
Fluid schematic for the DOT eDNA sampler. Nine filters are used for scheduled collection of samples by filtering 15 mL to 10 L or more of water, depending on the sample particulate loading. An inline pressure sensor is used to monitor transmembrane pressure to detect material accumulation on the filter membrane. RNAlater, 5% HCL, and Milli-Q water have routing paths used for preservation and cleaning.

The fluid schematic of the eDNA sampler is illustrated in Fig. 2. The eDNA sampler features several solenoid valves, filter membranes, onboard chemicals, and access to the surrounding fluids via the sample inlet and outlet ports. The eDNA sampler features custom control scripts that are used to coordinate operations between the solenoid valves and syringe pump. This coordination allows for fluid to be moved from one location of the sampler to another. The movement of fluid is performed concurrently with the monitoring and logging of both fluorometer and pressure readings. The custom scripts can be programmed into the eDNA sampler’s flash memory, or SD card storage, or manually entered over a terminal for greater control. The script that was used for this paper was one that was manually entered over the terminal to give the user greater control and debug visibility due to this being the first time the sampler was being deployed. The custom script contains the sampling protocol which sets the following: number of active valves, collection volume, time limit and minimum flow rate for each of its steps.

**Figure 3:**
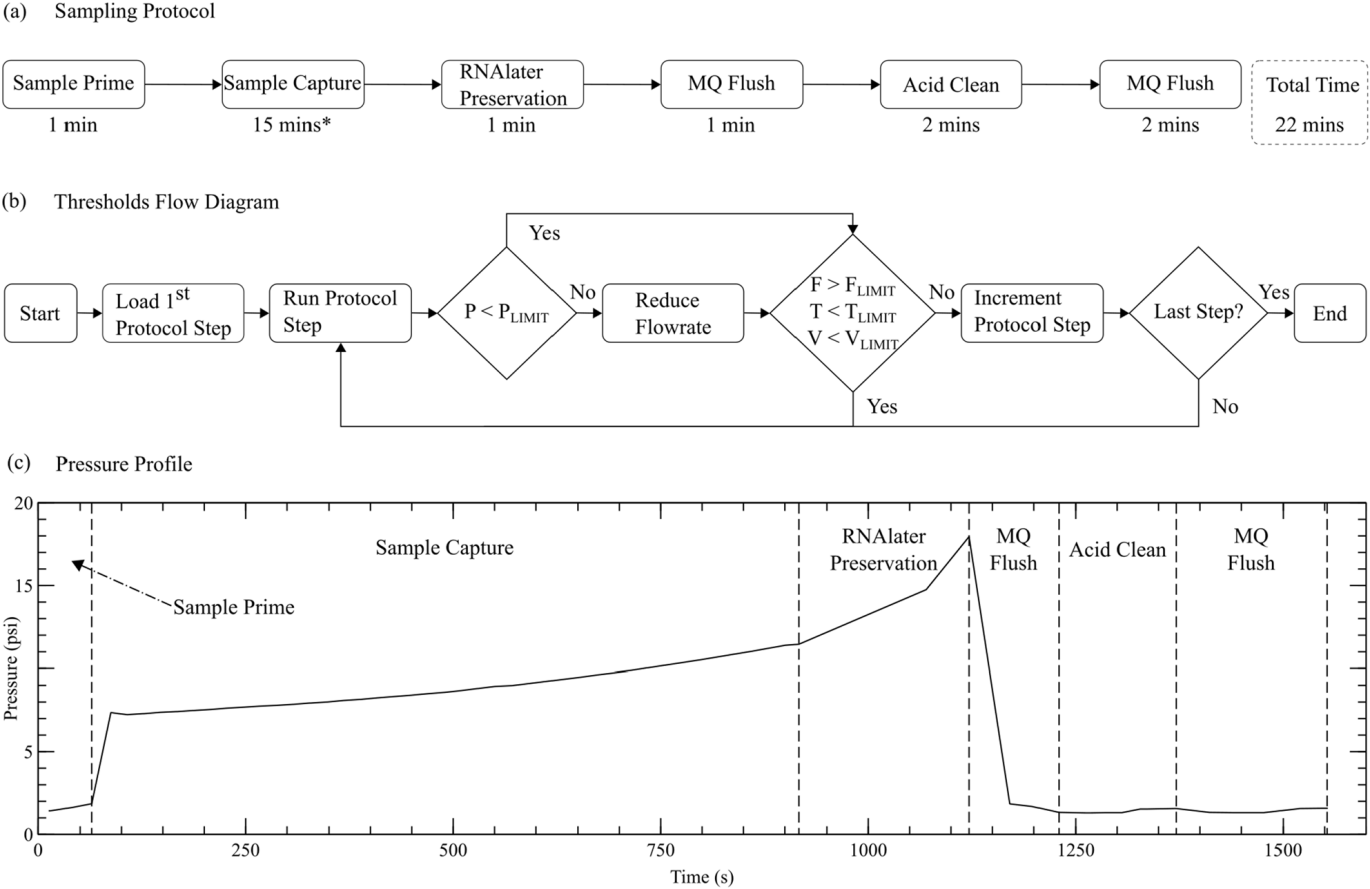
(**a**) Protocol used to capture and preserve sample then clean fluid channels. (**b**) Thresholds flow diagram used to load and run protocols within a safe user-specified operating region (F - Flow rate, P - Pressure, T - Time, V - Volume). (**c**) Pressure data captured during the sampling process on a 0.22 $\upmu$m polycarbonate filter membrane. * The sampling time is dependent on protocol-specified flow rate and fluid turbidity. The time shown above is for 20 ml/min in ideal conditions.

The sampling protocol used for the deployment described in this paper is shown in Fig. 3a. Below each step, the estimated completion time is shown. The “Sample Prime” step commences the sampling protocol and prepares the sampler by flushing its internal channels with the intended sample fluid. Thereafter, the sampler is now ready to perform the “Sample Capture” step. This step pushes the sample fluid through the selected filter membrane (M1 through M9) for sample capture. To preserve the material collected on the filter, the “RNAlater Preservation” step pushes the RNAlater through the selected filter membrane. The “MQ Flush” step then uses Milli-Q to flush RNAlater from the system to avoid it being in contact with 5% HCl that is used in the next step. The “Acid Clean” step cleans the sampler’s internal fluidic channels of contaminant, by using 5% HCl. Next, the “MQ Flush” step flushes the 5% HCl from the channels using Milli-Q. This process cleans the sampler and prepares it for the next sample capture. There is the possibility that residual acid will be left near the sample intake after backflowing the sample inlet with acid. However, after the acid backflush, the default protocol also pushes 9 ml of Milli-Q through the sample inlet to flush the lines and inlet of acid. This will force the dilute 5% HCl further away from the sample inlet and permit convective flow to remove localized acid before the next sampling event. In this deployment, the acid flush was at the end of a sample event and a minimum of 30 minutes between successive samples was used. In future, a minimum waiting period could be added to the protocol for low-flow or stagnant waters to prevent a false negative, where HCL would digest the sample in the environment prior to capture.

The algorithm shown in Fig. 3b is executed whenever sample capture is triggered. This algorithm runs the steps shown in the sampling protocol and monitors volume, pressure and time to ensure that the sampler stays within a tolerable running condition. The algorithm starts by running a series of checks. The first check is to determine that the pressure within the system does not exceed a preset pressure limit. If the pressure is greater than that limit the system reduces the flow rate by a preset 40%. After this the system checks the flow rate, time elapsed and volume since the start of the protocol step. If any of the limits are exceeded the sampler moves to the next step in the script. This procedure then repeats until there are no steps left in the sampling protocol. The sampler then enters a low-power state and waits for an interrupt to trigger the sampling protocol once more. Figure 3c illustrates the pressure profile of a recent eDNA sampler deployment in which the sampling protocol shown in Fig. 3a was performed.

The sampler is autonomous since it can perform all functionalities after being programmed with a sample schedule without the need for human or user interaction. The functionalities include sample capture, self-cleaning and sample preservation. The only human interaction is to change the filter cartridge, chemicals and battery in addition to programming the scheduler. Beyond scheduled triggering, the eDNA sampler contains an onboard 32-bit processor that allows it to be triggerable from external sensors and computers (e.g. AUV backseat systems).

### System architecture

**Figure 4:**
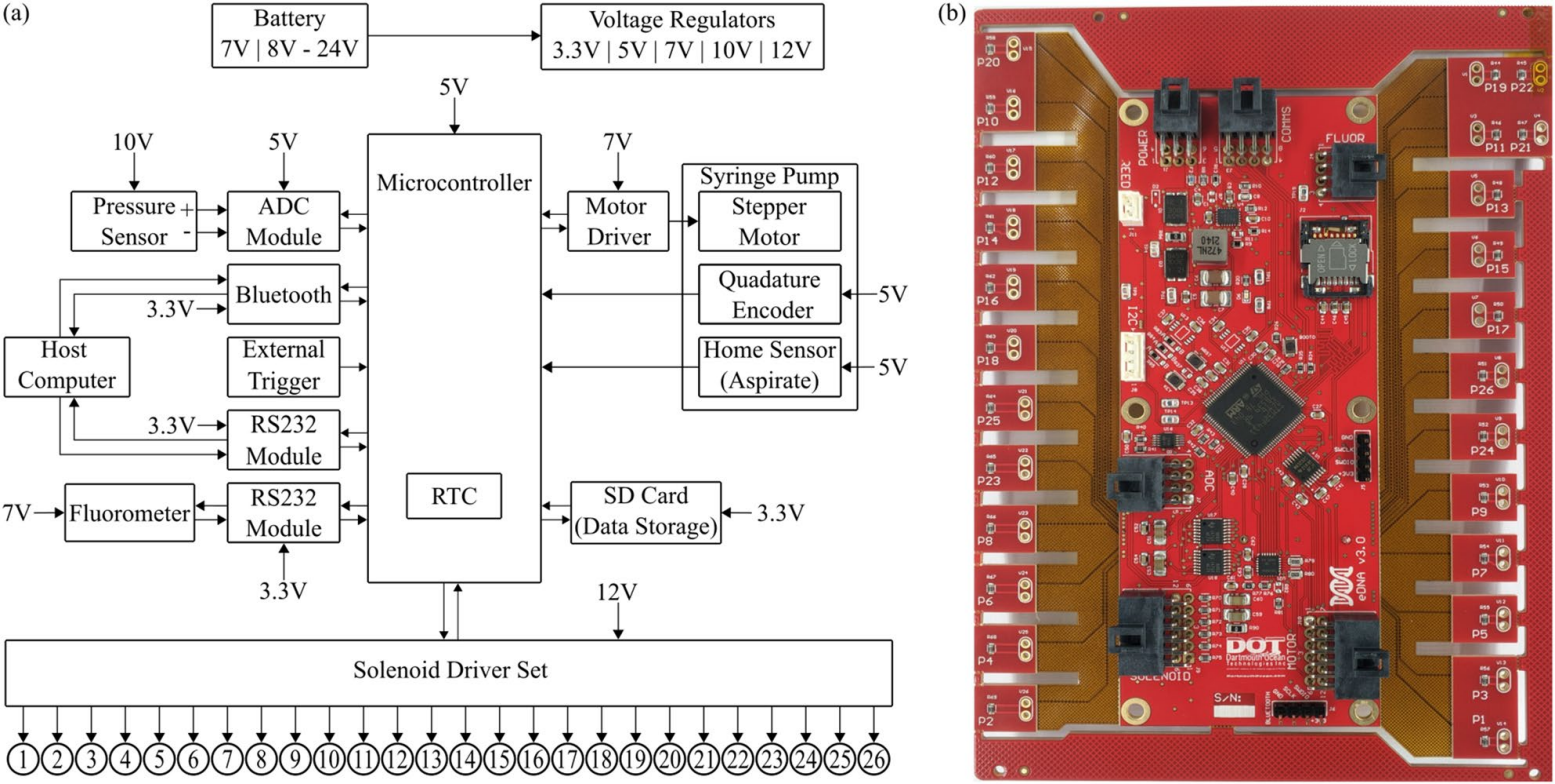
(**a**) Architecture diagram for the DOT eDNA sampler showing internal electrical connections and components along with interfaces to the external world, based on the STM32F411 microprocessor. (**b**) Front View of the designed PCB with surface mount components.

The eDNA sampler’s system architecture is shown in Fig. 4a. Figure 4b shows a fully built PCB for the eDNA sampler. Due to the varied electrical requirements of the sub-components, the system has regulators for generating multiple voltages ranging from 3.3 to 12 VDC, all sourced by a battery or power supply input of 7–24 VDC. The wide range of voltage input allows for flexibility in the platforms used for deployment (UAV/USVs, Buoys, Moorings, etc.). The system is controlled using an ARM Cortex M4 microcontroller (STM32F411) running at 84 MHz and configured as a Real Time System (RTS) that is governed by several interrupts and low-level controls to ensure precise timing. The single microcontroller manages the syringe pump, data logging, communication, and protocol execution. The syringe pump (an acid-tolerant custom variant of the LPDA1750330H, Lee Company Ltd.) is powered with a stepper-motor driver circuit (DRV8834, Texas Instruments) along with an optical quadrature encoder to aid the precise tracking of the volume used. The 26 solenoid valves used by the system are driven by a spike-and-hold circuit (DRV8860, Texas Instruments) that allows powering 32 valves without excessive current load. The DRV8860 is a serial connectable device and allows for a modular design for the expansion of solenoids that can be used by the system. The eDNA sampler makes use of a 16–bit ADC (ADS1115, Texas Instruments) module that is able to read the pressure sensor in a wheatstone bridge configuration with its builtin programmable gain amplifier (PGA). The amplified signal permits differential trans-membrane pressure measurements to be read to ensure that the membranes are used within the manufacturer’s specifications (typically under 4 bar, 60 psi). An optional pressure sensor can be added to read the ambient pressure of the environment and the depth of the sampler. The sampler stores all data on an internal 32 GB microSD card with timestamped files and folders. Users interface with the eDNA sampler through either Bluetooth (via a smartphone application) and/or through RS-232 and a personal computer terminal. These both permit operational commands to be sent to the sampler and are also conduits for transferring data to/from the system; for example, setting scheduled sampling times via the real-time clock (RTC) and/or for retrieving pressure and fluorometer data per membrane/sample. At idle the system draws 1 W, while sampling it draws 10 W peak.

The eDNA sampler was powered by a battery for the deployment described in this work. The single battery pack, 561.6 Wh Lithium thionyl chloride, lasted the entire deployment period. The battery can support a maximum of 33.7 L of pumping, assuming no blockage of the membranes, which should be sufficient for 32 filter captures at 1 L per capture and at 10 mL/min average flow rate. The litres pumped is the most appropriate metric for stating battery life expectations, as the pump consumes the most electricity in the eDNA sampler (8 W, 80 % of the power budget at peak). These assumptions also depend on the turbidity/particulate loading of the water when sampling. Beyond battery or power consumption, fluids are currently the limiting factor for continual use as the standard reagent reservoirs are good for 1 filter cartridge (9 filter captures), with plans to have an enhanced fluid capacity version that would support 3 filter cartridges (27 filter captures).

While we did not need an external power source for this demonstration, the eDNA sampler can also be powered from a typical 7 to 24 V DC supply. The power is provided through a 6-pin Subconn cable and can be supplied by a vehicle or platform. Given the low power consumption of the eDNA sampler (10 W peak), the sampler is highly amenable to the hotel load available on most autonomous vehicles and also solar powered buoys or installations.

### Field sample collection

**Figure 5:**
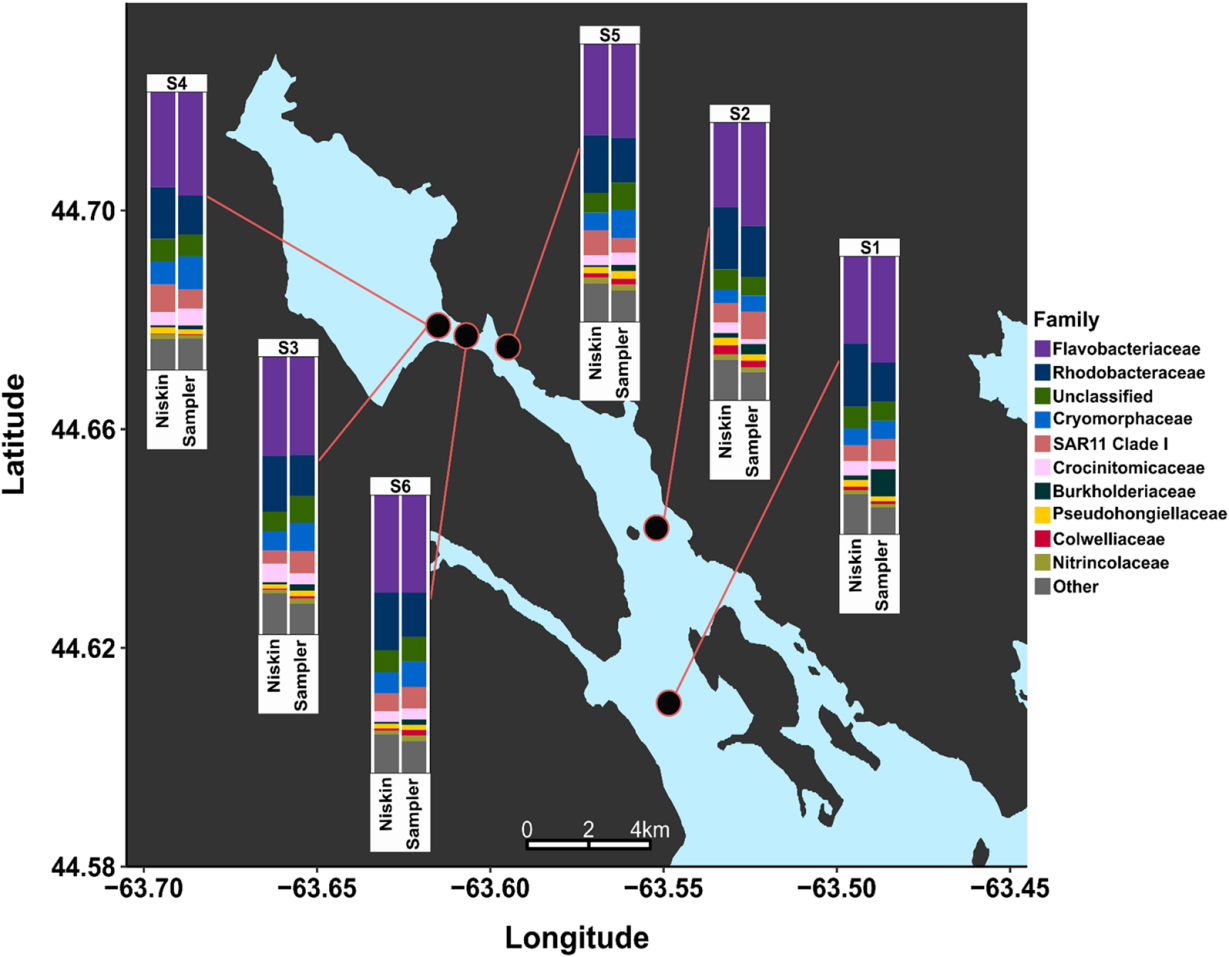
Map of Halifax Harbour sampling locations. Stations are numbered in sequential order with S1 being first and S6 last. Samples at S3 and S4 were taken at the same location approximately 2 hours apart. Stacked bar plots highlight the top 10 relatively abundant bacterial taxonomic families at each sampling station for all samples. All ASVs not within the top 10 families are represented as “Other”. The map presented here was created in RStudio^36^ using GADM data (Version 3.6)^48^ and R packages ggplot2^39^ and ggsn^49^.

As a first test of how this eDNA sampler performs in situ, the sampler was deployed during a transect of the Halifax Harbour into the Bedford Basin, shown in Fig. 5. Sampling was conducted along a series of stations for this transect in the Bedford Basin, where each station was sampled once. At each station, the unit was deployed 5 m deep and the first portion of the script was run, where a single sample of 125 mL of water was filtered through a 25 mm diameter, 0.22 $\upmu$m polycarbonate (PC) membrane (Millipore). The sampler was deployed for the entire time the boat was on station (15–17 min), ensuring the full 125 mL was captured. After pulling the sampler back on deck, the second portion of the script was run, where 6 mL of RNAlater was pumped across the membrane for preservation and the “Acid Clean” step was performed. Due to the time constraints of maintaining station, this 2-step approach was implemented; however, both steps are trivial to combine when the sampler is deployed as intended and without human intervention. Samples were stored in RNAlater in the cassette overnight, after which they and the 35 $\upmu$m pre-filter were removed and stored short-term in a −20 $^{\circ }$C freezer, then longer term in a −80 $^{\circ }$C freezer prior to extracting the DNA. Though the sampler preserves the samples with RNAlater automatically, filters were frozen as recommended for long term storage due to the unknown timeline between the transect and DNA extraction. For this deployment, the sampler included a 35 $\upmu$m pre-filter on the inlet. This inlet filter was added because in the current setup, the valves are not rated for particles over 35 $\upmu$m. The pre-filter may limit the sampler to applications studying micro-organisms with cell sizes smaller than 35 $\upmu$m. We are currently investigating the system performance with a larger 100 $\upmu$m pre-filter.

**Figure 6:**
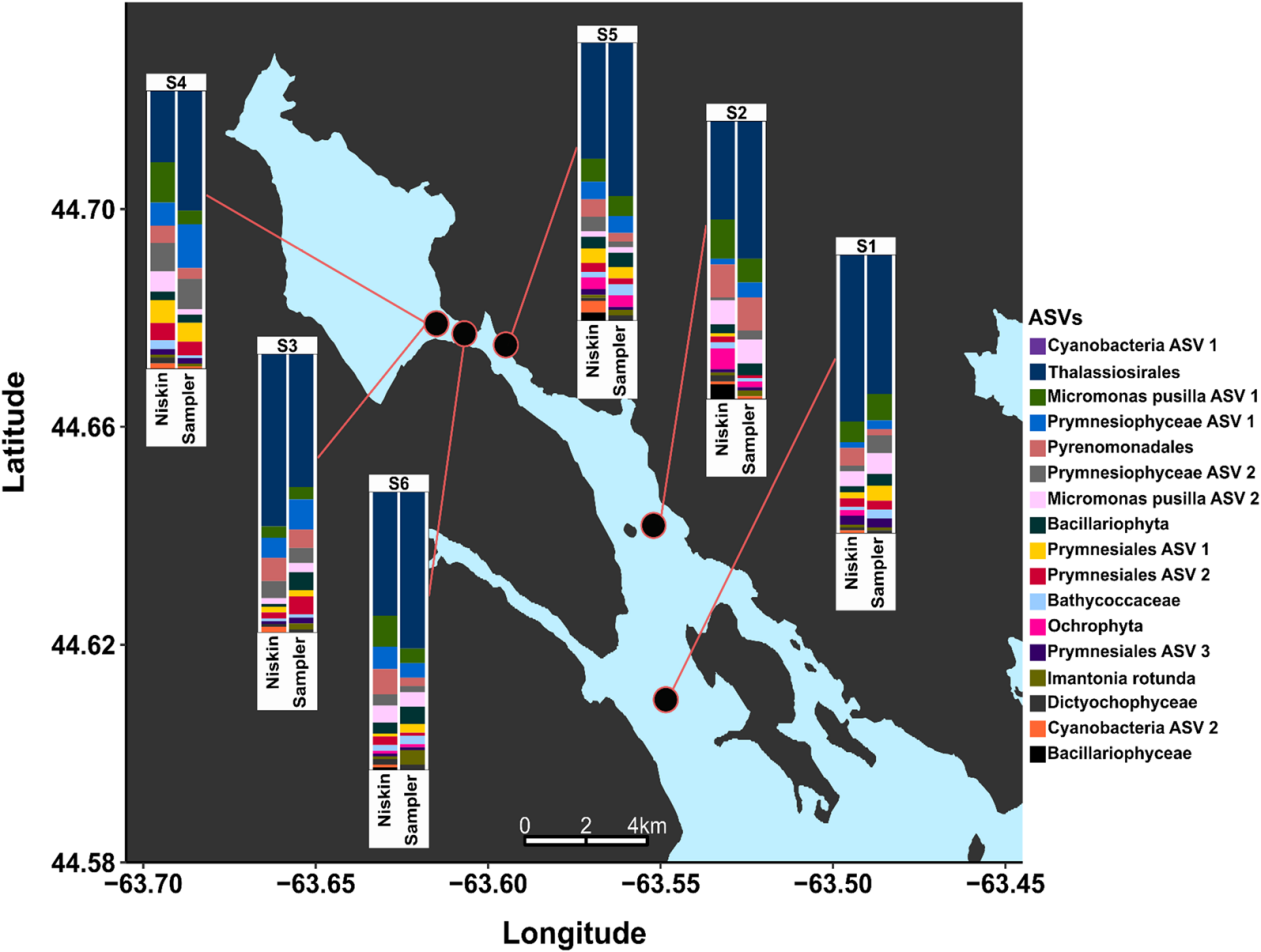
Map of Halifax Harbour sampling locations. Stations are numbered in sequential order with S1 being first and S6 last. Samples at S3 and S4 were taken at the same location approximately 2 hours apart. Stacked bar plots were created by identifying the top 10 relatively abundant chloroplast 16S rRNA ASVs in each sample. ASVs are identified down to the lowest taxonomic rank possible, and ASVs with the same taxonomy are further distinguished as “ASV 1” through “ASV 3”. The first ASV in the legend, labelled as “Cyanobacteria ASV 1”, is the only ASV also recovered from the pre-filter. The map presented here was created in RStudio^36^ using GADM data (Version 3.6)^48^ and R packages ggplot2^39^ and ggsn^49^.

Parallel bottle samples were taken at the same time and depth at each station using a 5 L Niskin bottle attached to a rope. At each station the Niskin was deployed to capture a water sample directly next to the sampler. This water sample was then divided into two bottles, which were both filtered on deck using a peristaltic pump through 47 mm diameter, 0.22 $\upmu$m PC membranes (Millipore), resulting in duplicate filters from each Niskin deployment. Between 660 and 1140 mL of water was filtered for each duplicate, after which the volume filtered was recorded and membranes were immediately frozen in a cryoshipper primed with liquid nitrogen.

### DNA extraction and sequencing

DNA from all sampler filters and one Niskin duplicate from each station were extracted and processed. The other Niskin samples were archived and preserved at −80 $^{\circ }$C as a backup in case there were issues with extraction or sequencing. The Qiagen DNeasy plant mini kit was used to extract DNA from all samples using a modified protocol based on Zorz et al.^25^ with the following additional modifications: samples were incubated at 52 $^{\circ }$C for 1 hour and the same 50 $\upmu$l of elution buffer from Qiagen (AE buffer) was used to elute the DNA twice to ensure maximum DNA concentrations were extracted. After extraction, 10 $\upmu$l of DNA from each sample was sent for Illumina sequencing at the Integrated Microbiome Resource Lab (IMR) at Dalhousie University. DNA was sequenced for the V4-V5 region of the 16S ribosomal RNA gene according to IMR standard operating procedure for amplicon sequencing as outlined in Comeau et al.^26^ . 16S amplicon fragments were amplified in duplicate using a single round of PCR using fusion primers (Illumina adaptors + indices + specific regions) 515F = 5′-GTGYCAGCMGCCGCGGTAA-3′ and 926R = 5′-CCGYCAATTYMTTTRAGTTT-3′^27,28^.

### Bioinformatics

Sequences were processed according to the Microbiome Helper developed by IMR^26^ using QIIME2 2019.7^29^. Deblur (QIIME2 plugin version 2019.7)^30^ was used to denoise sequences as well as identify and label individual amplicon sequence variants (ASVs)^31^. ASVs are clusters of highly related DNA sequences that are treated as a single homogeneous unit; each is assigned to a particular taxonomic group such as species, with multiple ASVs potentially mapping to the same group. After identification, ASVs were classified using the SILVA 132 database^32,33^ as well as the PhytoREF database^34^ for further classification of chloroplast sequences. Two ASV tables were created from this data: one with the raw ASV counts, and a second where raw ASV counts were rarefied to 4000 so relative abundance could be compared between samples. Rarefaction is a normalization process through which samples of differing sizes are subsampled to a normalized threshold^35^. Rarefaction curves in Fig. S2 can be found in the supporting information. All plots were made using RStudio^36^ using the following packages: UpSetR^37^, Phyloseq^38^, ggplot2^39^, and ggpmisc^40^.

## Results

### DNA extraction and sequencing

In total, 6 stations were sampled in the first field deployment of the eDNA sampler; the coordinates and the time at which samples were taken can be found in Table S1. The map of stations sampled is shown in Fig. 5. At 2 out of the 6 stations, S2 and S6, the preset target volume was not reached. This was due to the transmembrane pressure reaching the threshold, with enough material accumulated to block the filter membrane, therefore the time threshold of staying on the station was met before the volume threshold. The remaining stations, S1, S3, S4, and S5 had 100% of the sample captured, as shown in Table 2. Table 2 outlines various metrics regarding the extracted DNA and raw sequencing data for each transect sample and the pre-filter. Although eluted DNA concentrations were lower in samples collected using the sampler, when considering the difference in volume filtered, the original source DNA concentration (i.e. marine water) is comparable between the Niskin and sampler, but not identical as DNA extractions are not 100% efficient. As well, the pre-filter had a lower eluted DNA concentration and nearly 0 ng/mL in the original sample.

**Table 2:** DNA metrics of eDNA sampler and Niskin samples at each station (S1–S6) and the pre-filter (PF). DNA in the original sample was back calculated from the concentration of eluted DNA using the elution volume (50 $\upmu$L for all samples). The 260/280 ratio is presented as an indicator of purity. While 260/280 ratio values are expected to be 1.8 for DNA and 2.0 for RNA, the actual ratio is a factor of the nucleic acid composition as well as the pH of the solution^41^.

Station	Method	Sample volume (mL)	Eluted DNA (ng/$\upmu$L)	DNA in sample (ng/mL)	260/280 ratio	Number of raw reads	Number of ASVs
S1	Sampler	125	8.3	3.3	2.23	37081	278
	Niskin	760	97.3	6.4	1.91	5511	187
S2	Sampler	111	4.9	2.2	2.05	46863	304
	Niskin	740	57.8	3.9	1.9	5404	193
S3	Sampler	125	10.6	4.2	2.15	30270	282
	Niskin	770	93.8	6.1	1.86	11839	225
S4	Sampler	125	16.4	6.6	2.05	20218	242
	Niskin	660	55.1	4.2	1.89	6901	191
S5	Sampler	125	4.7	1.9	2.27	10675	233
	Niskin	880	35.5	2.0	1.92	8972	223
S6	Sampler	114	9.4	4.1	2.15	12667	238
	Niskin	1140	68.5	3.0	1.92	14000	240
PF	Sampler	875	3.1	0.2	2.48	25100	255

### Community composition

Figure 5 shows the 10 families with the highest relative abundance at each station for both collection methods, also shown side-by-side in Fig. S1. The two collection methods returned the same top families in near-identical relative abundance, demonstrating that the sampler was able to capture the same community composition of common microbes as the Niskin. All of the 10 most-abundant families were found in all 12 samples, with Rhodobacteraceae having the greatest difference in relative abundance between Niskin and sampler at 5 out of 6 stations (9% in S1, 5% in S3–S6) and Flavobacteraceae having the greatest difference in S2 (6%). The difference between all other taxa at each station was less than 5% with the exception of Burkholderiaceae. The family Burkholderiaceae showed high variance at site S1 (sampler: 10%, Niskin: 2%) and to a lesser extent S2 (sampler: 4%, Niskin: 2%). This was due to a particular ASV classified as *Ralstonia picketti*, which was found in all of the sampler samples, but none of the Niskin samples. A similar analysis of the phytoplankton community in Fig. 6 shows a strong bloom of Thalassiosirales which presented as a single chloroplast ASV dominating both sample types (Niskin and sampler) at all stations, ranging from 30% of all chloroplast reads in S4 Niskin to 60% in S3 Niskin. No evidence of Thalassiosirales was found in the pre-filter sample and the only ASV found in the pre-filter (represented as Cyanobacteria in Fig. 6) was found in low relative abundance in the rest of the samples.

**Figure 7:**
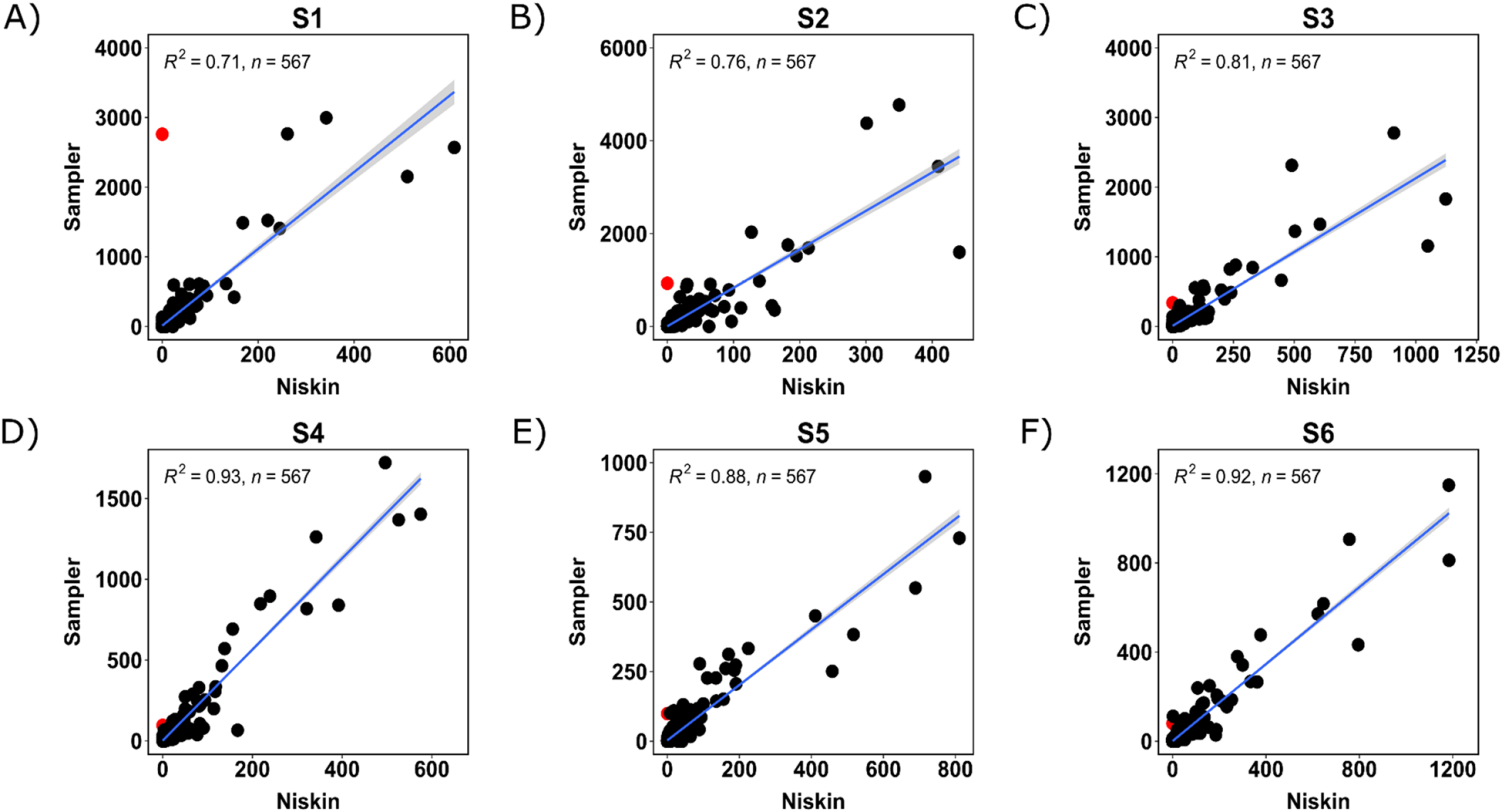
Scatterplots showing raw counts of all ASVs in samples from each collection method plotted against each other. The single red point indicates the raw counts of *Ralstonia picketti*, a potential contaminant found only in the sampler. The contaminant decreases with more utilization of the sampler, from S1 to S6, indicating that new sampler builds must be thoroughly cleaned after assembly to remove contaminants.

The Niskin and eDNA sampler results were also quantitatively similar at the level of individual ASVs. Figure 7 shows the correlation between sampler ASVs and Niskin ASVs (both bacterial and phytoplankton) at each site. R$^{2}$ values ranged from 0.71 to 0.93, with later stations having higher R$^{2}$ values than stations S1 and S2. The *Ralstonia picketti* ASV mentioned previously is highlighted in red, and has higher counts in S1 and S2 (7.5% and 2% of total counts respectively), with lower abundance in stations S3–S6 ($\le$ 1% of total counts). Figure S3 depicts a scatterplot of the combined counts of each ASV across stations 1–6 for each method.

**Figure 8:**
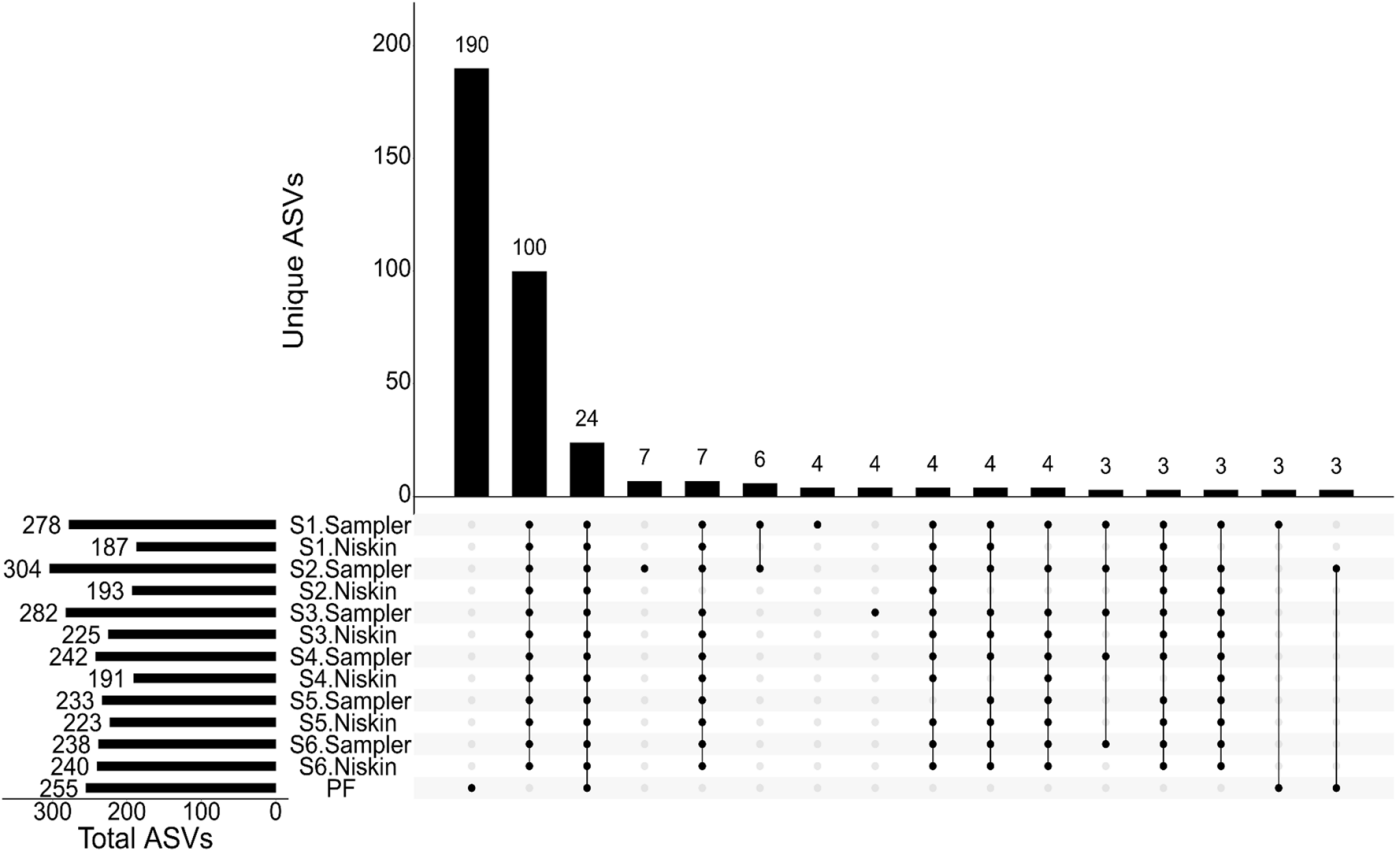
Upset plot of ASVs in each sample as well as the pre-filter (PF). Each column shows the count of ASVs with the occurrence pattern indicated by the black dots with the associated samples. The total number of ASVs associated with each sample is shown on the left. Sets of samples with 3 or more associated ASVs are shown here, with sample combinations returning ASVs occurring only once or twice not shown.

Lastly, we examined the patterns of presence and absence of each ASV across the 13 sampler, Niskin, and pre-filter samples. The most common presence / absence pattern included 190 ASVs that were found only in the pre-filter sample (Fig. 8); however, these account for less than 10% (20,936 of 235,501 sequences collected from all samples). By contrast, only 65 ASVs were found in the pre-filter and at least one other sample. This further reinforces that the pre-filter did not filter out any important ASVs in the water column, and instead had its own composition. A total of 124 ASVs were found in all twelve Bedford Basin sample sites, 24 of which were also recovered from the pre-filter. Of these, the 100 ASVs recovered only from Bedford Basin sites accounted for 171,345 sequences (72.8%) while the 24 ASVs recovered from all samples accounted for 23,341 sequences (10.0% of all recovered sequences). No other presence / absence pattern was exhibited by more than seven ASVs, and the majority of patterns were observed once or twice in the pool of ASVs. These results further demonstrate the homogeneity of the samples across stations and the consistency between the sampler and Niskin datasets. A total of 4308 sequences were assigned to the probable contaminant *Ralstonia pickettii* across all eDNA sampler samples; these decreased in count from 4308 in sample S1 to 80 in the final sample S6.

## Discussion

The results presented in this paper detail the successful testing and deployment of a novel eDNA sampler. When compared to the concurrent Niskin bottle samples, the sampler captured a near-identical community for both bacteria and phytoplankton at all stations. This is despite differences in the protocol such as volume filtered and temporal resolution, aligning with prior studies, which have shown similar results^42,43^. A recent study performed using the 3G ESP also demonstrated that results were equivalent between autonomous and manual sampling^44^. Interestingly, sample volumes in the ESP study were reversed where the autonomous sampler filtered a higher volume (1 L), and the manual sampling filtered a lower volume (36–100 mL)^44^. Our protocol generated comparable results with a lower autonomous sampler volume, which allows sampling time to be kept to a minimum.

Another difference between the sampler and Niskin methods is the 35 $\upmu$m pre-filter fitted on the inlet of the sampler due to particle limitations on the pump and valves. However, results here show that the pre-filter did not affect results as the sampler still picked up the community in the water column. Keeping the pre-filter is advantageous because it allows the sampler to remain at a small, portable size, allowing for easier field deployment, particularly on small vessels with little deck space. The pre-filter also did not affect results through clogging, due to the cleaning protocol and pre-sample flushes which include a backflush through the inlet, thereby pushing material off the pre-filter.

The one noticeable discrepancy between samples was that an ASV classified as *Ralstonia picketti* was found in all sampler results but none of the Niskin results. This bacterium is commonly found in the environment and is capable of growing on plastics^45^, meaning it was likely a form of contamination in the sampler from previous testing. Despite the prevalence of this bacteria, the raw counts and relative abundance decreased rapidly in subsequent sampling, indicating the cleaning protocol was clearing the bacterium out of the lines with each sample. Therefore, with optimization, the cleaning protocol can prevent contamination in the future.

In this work, negative controls were not used. This is due to the Niskin bottle captures acting as a control or comparison mechanism. However, the system can be configured in such a way to utilize the on-board Milli-Q reserves as a negative control mechanism. Assuming volume of reagents are not a constraint, one mode of operation could be to have a Milli-Q blank between every sample, or 5 samples and 4 blanks. The drawback to this is the potentially large volumes of Milli-Q (blank/negative control) that would need to be deployed and replaced after each deployment.

In this study, each site was sampled once with the eDNA sampler and the Niskin Bottle capture. However, past publications demonstrate that the bacterial composition can change on a weekly time scale in the Bedford Basin^46^. Now that it has been shown that the initial eDNA sampler configuration can be successfully deployed, time-series studies in a variety of aquatic environments would enable insight into microbial changes with minimal human involvement. Beyond time-series studies, deploying multiple samplers in replicate can also be performed to evaluate inter-instrument repeatability. Both these types of studies are currently planned for 2023–2024 and will see 6 samplers deployed over multiple months.

Future studies could also be used to look at eRNA, in order to make use of the advantage that the sampler preserves nucleic acids immediately after sampling. Although we observed excellent performance with RNAlater, testing different chemicals for preservation would be beneficial, as there are many labs that use other solutions such as Longmire’s buffer^47^ and DNAgard$\circledR$^16^ to preserve their samples.

In conclusion, these results demonstrate the successful testing and deployment of a novel, autonomous eDNA sampler capable of both cleaning and preservation, and includes a field-swappable cartridge. These aspects are beneficial for research and monitoring, particularly in remote locations and over long periods of time in areas such as Marine Protected Areas (MPAs). Future studies of the system will include cleaning optimization as well as fluorometer integration and collaborative testing of the system in multiple deployment scenarios. Overall, this eDNA sampler will expand the use of eDNA in monitoring programs, making it more accessible and convenient than traditional sampling methods.

## Supplementary Information

Supplementary Information.

## Data Availability

The datasets generated and/or analysed during the current study are available in the National Center for Biotechnology Information repository, https://www.ncbi.nlm.nih.gov/sra/PRJNA917080.
