# Supplementary material for: Compact and automated eDNA sampler for in situ monitoring of marine environments
Source: Sci Rep. 2023 Mar 30;13:5210. doi: 10.1038/s41598-023-32310-3 (PMC10063616; doi:10.1038/s41598-023-32310-3)
Supplement: Supplementary file 1 — Supplementary Information. [file 41598_2023_32310_MOESM1_ESM.pdf]

Supplementary Material to "Compact and Automated eDNA Sampler for in situ Monitoring of Marine Environments"

Andre Hendricks[1,+,*], Connor M. Mackie[2,+], Edward Luy[2], Colin Sonnichsen[1,2], James Smith[2], Iain Grundke[2], Mahtab Tavasoli[3], Arnold Furlong[2], Robert G. Beiko[4,*], Julie LaRoche[5,*], and Vincent Sieben[1,2,*]

[1]Department of Electrical and Computer Engineering, Dalhousie University, Halifax, Nova Scotia, Canada
[2]Dartmouth Ocean Technologies Inc, Dartmouth, Nova Scotia, Canada
[3]Department of Pharmacology, Dalhousie University, Halifax, Nova Scotia, Canada
[4]Faculty of Computer Science, Dalhousie University, Halifax, Nova Scotia, Canada
[5]Department of Biology, Dalhousie University, Halifax, Nova Scotia, Canada
[*]Andre.Hendricks@dal.ca; rbeiko@dal.ca; julie.laroche@dal.ca; sieben@dal.ca
[+]These authors contributed equally to this work

ABSTRACT

Using environmental DNA (eDNA) to monitor biodiversity in aquatic environments is becoming an efficient and cost-effective alternative to other methods such as visual and acoustic identification. Until recently, eDNA sampling was accomplished primarily through manual sampling methods; however, with technological advances, automated samplers are being developed to make sampling easier and more accessible. This paper describes a new eDNA sampler capable of self-cleaning, and multi-sample capture and preservation, all within a single unit capable of being deployed by a single person. The first in-field test of this sampler took place in the Bedford Basin, Nova Scotia, Canada alongside parallel samples taken using the typical Niskin bottle collection and post-collection filtration method. Both methods were able to capture the same aquatic microbial community and counts of representative DNA sequences were well correlated between methods with R^2 values ranging from 0.71–0.93. The two collection methods returned the same top 10 families in near identical relative abundance, demonstrating that the sampler was able to capture the same community composition of common microbes as the Niskin. The presented eDNA sampler provides a robust alternative to manual sampling methods, is amenable to autonomous vehicle payload constraints, and will facilitate persistent monitoring of remote and inaccessible sites.

1 Supplementary Information

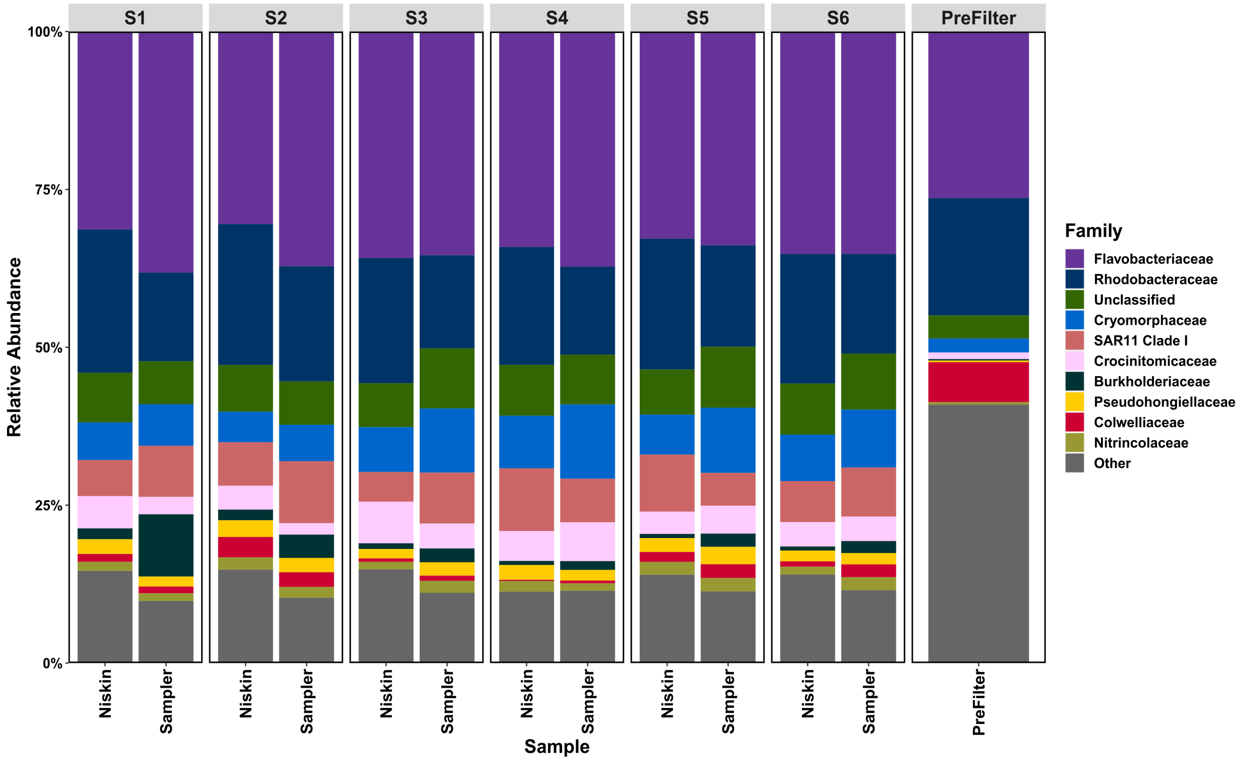

Figure S1. Stacked bar plot highlighting the top 10 relatively abundant bacterial taxonomic families at each sampling station for all samples as well as the pre-filter. ASVs were rarefied to 4000 to obtain relative abundance and all ASVs not within the top 10 families are represented as "Other".

Table S1. Coordinates and time sampled for each of the 6 stations where a successful deployment occurred. S3 and S4 represent samples taken at the same location at different times.

Station	Coordinates	Time Sampled
S1	44.36947 °N, 63.32909 °W	10:15
S2	44.640941 °N, 63.552115 °W	11:15
S3	44.680485 °N, 63.622483 °W	12:45
S4	44.680485 °N, 63.622483 °W	14:40
S5	44.674603 °N, 63.595273 °W	15:15
S6	44.670648 °N, 63.592853 °W	16:02

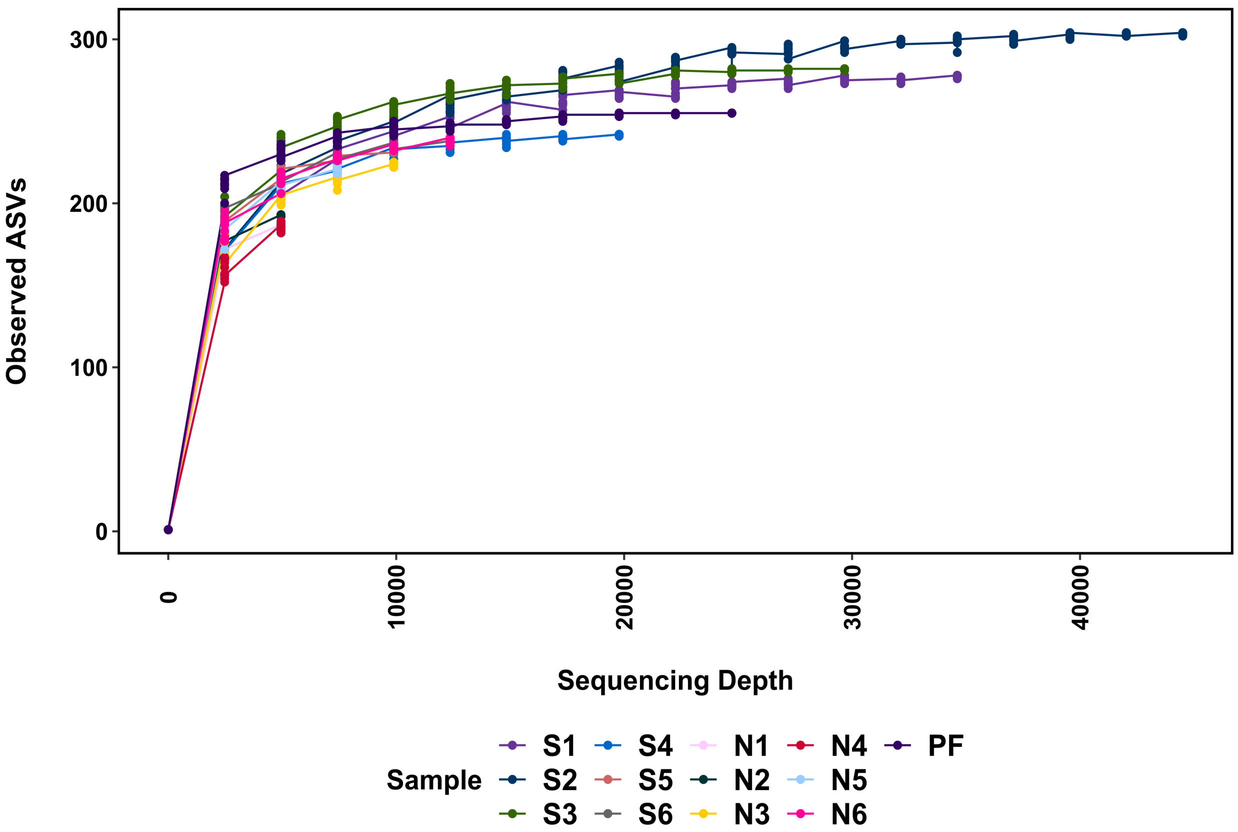

Figure S2. Rarefaction curves for all samples. S1 to S6 are the samples acquired from the eDNA sampler, while N1-N6 are the samples acquired from the Niskin bottle captures. PF is the 35 micron pre-filter on the eDNA sampler.

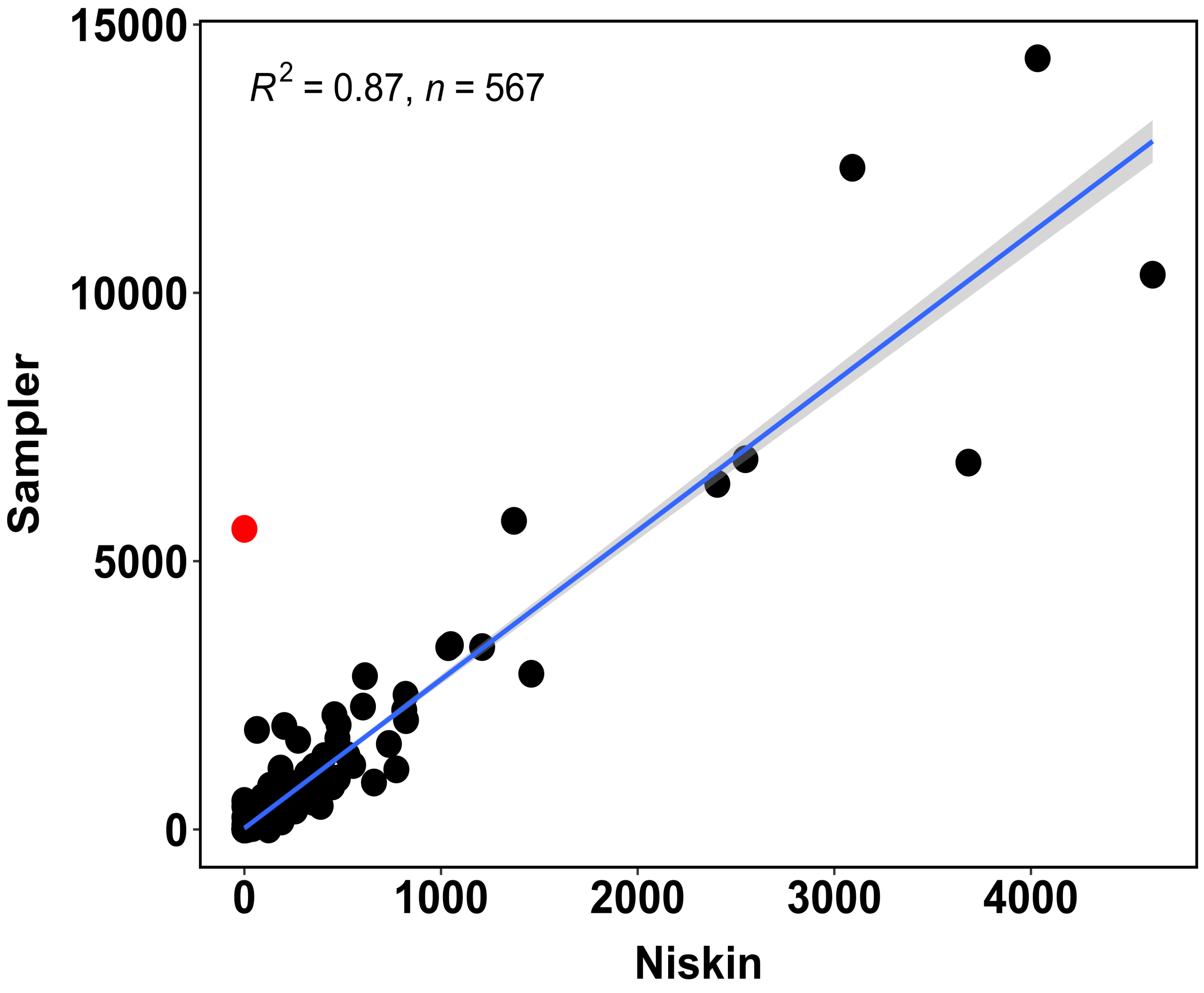

Figure S3. Scatterplot showing raw counts of all ASVs in all samples from each collection method plotted against each other. The single red point indicates the raw counts of *Ralstonia picketti*, a potential contaminant found only in the sampler as discussed in the manuscript.
